# Supplementary material for: Resolving the full spectrum of human genome variation using Linked-Reads
Source: Genome Res. 2019 Apr;29(4):635–45. doi: 10.1101/gr.234443.118 (PMC6442396; doi:10.1101/gr.234443.118)
Supplement: Supplemental Material [file supp_gr.234443.118_Supplemental_longranger-2.2.zip › longranger-2.2.tar.bz2!/lr2.2-public/lib/stan/cmdstan/stan/doc/api/html/pareto__type__2_8hpp_source.html]

Stan: src/stan/prob/distributions/univariate/continuous/pareto\_type\_2.hpp Source File


|  |  |
| --- | --- |
|  | Stan  2.5.0  probability, sampling & optimization |


- Main Page
- Related Pages
- Namespaces
- Classes
- Files

- File List
- File Members

All Classes Namespaces Files Functions Variables Typedefs Enumerations Enumerator Friends Macros Pages

- src
- stan
- prob
- distributions
- univariate
- continuous

pareto\_type\_2.hpp

Go to the documentation of this file.

1 #ifndef STAN\_\_PROB\_\_DISTRIBUTIONS\_\_UNIVARIATE\_\_CONTINUOUS\_\_PARETO\_TYPE\_2\_HPP

2 #define STAN\_\_PROB\_\_DISTRIBUTIONS\_\_UNIVARIATE\_\_CONTINUOUS\_\_PARETO\_TYPE\_2\_HPP

3

4 #include <boost/random/variate\_generator.hpp>

5 #include <stan/prob/distributions/univariate/continuous/uniform.hpp>

6

7 #include <stan/agrad/partials\_vari.hpp>

8 #include <stan/math/error\_handling.hpp>

9 #include <stan/math/constants.hpp>

10 #include <stan/math/functions/value\_of.hpp>

11 #include <stan/math/functions/log1m.hpp>

12 #include <stan/meta/traits.hpp>

13 #include <stan/prob/constants.hpp>

14 #include <stan/prob/traits.hpp>

15

16

17 namespace stan {

18  namespace prob {

19

20  // pareto\_type\_2(y|lambda,alpha) [y >= 0; lambda > 0; alpha > 0]

21  template <bool propto,

22  typename T\_y, typename T\_loc, typename T\_scale, typename T\_shape>

23  typename return\_type<T\_y,T\_loc,T\_scale,T\_shape>::type

24  pareto\_type\_2\_log(const T\_y& y, const T\_loc& mu, const T\_scale& lambda,

25  const T\_shape& alpha) {

26  static const char\* function = "stan::prob::pareto\_type\_2\_log(%1%)";

27

28  using std::log;

29  using stan::math::value\_of;

30  using stan::math::check\_finite;

31  using stan::math::check\_greater\_or\_equal;

32  using stan::math::check\_positive\_finite;

33  using stan::math::check\_nonnegative;

34  using stan::math::check\_not\_nan;

35  using stan::math::check\_consistent\_sizes;

36

37  // check if any vectors are zero length

38  if (!(stan::length(y)

39  && stan::length(mu)

40  && stan::length(lambda)

41  && stan::length(alpha)))

42  return 0.0;

43

44  // set up return value accumulator

45  double logp(0.0);

46

47  // validate args (here done over var, which should be OK)

48  check\_greater\_or\_equal(function, y, mu, "Random variable", &logp);

49  check\_not\_nan(function, y, "Random variable", &logp);

50  check\_positive\_finite(function, lambda, "Scale parameter", &logp);

51  check\_positive\_finite(function, alpha, "Shape parameter", &logp);

52  check\_consistent\_sizes(function,

53  y,lambda,alpha,

54  "Random variable","Scale parameter",

55  "Shape parameter",

56  &logp);

57

58  // check if no variables are involved and prop-to

59  if (!include\_summand<propto,T\_y,T\_scale,T\_shape>::value)

60  return 0.0;

61

62  VectorView<const T\_y> y\_vec(y);

63  VectorView<const T\_loc> mu\_vec(mu);

64  VectorView<const T\_scale> lambda\_vec(lambda);

65  VectorView<const T\_shape> alpha\_vec(alpha);

66  size\_t N = max\_size(y, mu, lambda, alpha);

67

68  // set up template expressions wrapping scalars into vector views

69  agrad::OperandsAndPartials<T\_y,T\_loc,T\_scale,T\_shape>

70  operands\_and\_partials(y, mu, lambda, alpha);

71

72  DoubleVectorView<include\_summand<propto,T\_y,T\_loc,T\_scale,T\_shape>::value,

73  is\_vector<T\_y>::value> log1p\_scaled\_diff(N);

74  if (include\_summand<propto,T\_y,T\_loc,T\_scale,T\_shape>::value)

75  for (size\_t n = 0; n < N; n++)

76  log1p\_scaled\_diff[n] = log1p((value\_of(y\_vec[n])

77  - value\_of(mu\_vec[n]))

78  / value\_of(lambda\_vec[n]));

79

80  DoubleVectorView<include\_summand<propto,T\_scale>::value,

81  is\_vector<T\_scale>::value> log\_lambda(length(lambda));

82  if (include\_summand<propto,T\_scale>::value)

83  for (size\_t n = 0; n < length(lambda); n++)

84  log\_lambda[n] = log(value\_of(lambda\_vec[n]));

85

86  DoubleVectorView<include\_summand<propto,T\_shape>::value,

87  is\_vector<T\_shape>::value> log\_alpha(length(alpha));

88  if (include\_summand<propto,T\_shape>::value)

89  for (size\_t n = 0; n < length(alpha); n++)

90  log\_alpha[n] = log(value\_of(alpha\_vec[n]));

91

92  DoubleVectorView<!is\_constant\_struct<T\_shape>::value,

93  is\_vector<T\_shape>::value> inv\_alpha(length(alpha));

94  if (!is\_constant\_struct<T\_shape>::value)

95  for (size\_t n = 0; n < length(alpha); n++)

96  inv\_alpha[n] = 1 / value\_of(alpha\_vec[n]);

97

98  for (size\_t n = 0; n < N; n++) {

99  const double y\_dbl = value\_of(y\_vec[n]);

100  const double mu\_dbl = value\_of(mu\_vec[n]);

101  const double lambda\_dbl = value\_of(lambda\_vec[n]);

102  const double alpha\_dbl = value\_of(alpha\_vec[n]);

103  const double sum\_dbl = lambda\_dbl + y\_dbl + mu\_dbl;

104  const double inv\_sum = 1.0 / sum\_dbl;

105  const double alpha\_div\_sum = alpha\_dbl / sum\_dbl;

106  const double deriv\_1\_2 = inv\_sum + alpha\_div\_sum;

107

108  // // log probability

109  if (include\_summand<propto,T\_shape>::value)

110  logp += log\_alpha[n];

111  if (include\_summand<propto,T\_scale>::value)

112  logp -= log\_lambda[n];

113  if (include\_summand<propto,T\_y,T\_scale,T\_shape>::value)

114  logp -= (alpha\_dbl + 1.0) \* log1p\_scaled\_diff[n];

115

116  // gradients

117  if (!is\_constant\_struct<T\_y>::value)

118  operands\_and\_partials.d\_x1[n] -= deriv\_1\_2;

119  if (!is\_constant\_struct<T\_loc>::value)

120  operands\_and\_partials.d\_x2[n] += deriv\_1\_2;

121  if (!is\_constant\_struct<T\_scale>::value)

122  operands\_and\_partials.d\_x3[n] -= alpha\_div\_sum \* (mu\_dbl - y\_dbl)

123  / lambda\_dbl + inv\_sum;

124  if (!is\_constant\_struct<T\_shape>::value)

125  operands\_and\_partials.d\_x4[n] += inv\_alpha[n] - log1p\_scaled\_diff[n];

126  }

127  return operands\_and\_partials.to\_var(logp);

128  }

129

130  template <typename T\_y, typename T\_loc, typename T\_scale, typename T\_shape>

131  inline

132  typename return\_type<T\_y,T\_loc,T\_scale,T\_shape>::type

133  pareto\_type\_2\_log(const T\_y& y, const T\_loc& mu,

134  const T\_scale& lambda, const T\_shape& alpha) {

135  return pareto\_type\_2\_log<false>(y,mu,lambda,alpha);

136  }

137

138  template <typename T\_y, typename T\_loc, typename T\_scale, typename T\_shape>

139  typename return\_type<T\_y, T\_loc, T\_scale, T\_shape>::type

140  pareto\_type\_2\_cdf(const T\_y& y, const T\_loc& mu,

141  const T\_scale& lambda, const T\_shape& alpha) {

142

143  // Check sizes

144  // Size checks

145  if ( !( stan::length(y)

146  && stan::length(mu)

147  && stan::length(lambda)

148  && stan::length(alpha) ) )

149  return 1.0;

150

151  // Check errors

152  static const char\* function = "stan::prob::pareto\_type\_2\_cdf(%1%)";

153

154  using stan::math::check\_greater\_or\_equal;

155  using stan::math::check\_finite;

156  using stan::math::check\_positive\_finite;

157  using stan::math::check\_not\_nan;

158  using stan::math::check\_greater\_or\_equal;

159  using stan::math::check\_consistent\_sizes;

160  using stan::math::check\_nonnegative;

161  using stan::math::value\_of;

162

163  double P(1.0);

164

165  check\_greater\_or\_equal(function, y, mu, "Random variable", &P);

166  check\_not\_nan(function, y, "Random variable", &P);

167  check\_nonnegative(function, y, "Random variable", &P);

168  check\_positive\_finite(function, lambda, "Scale parameter", &P);

169  check\_positive\_finite(function, alpha, "Shape parameter", &P);

170  check\_consistent\_sizes(function, y, lambda, alpha,

171  "Random variable", "Scale parameter",

172  "Shape parameter", &P);

173

174  // Wrap arguments in vectors

175  VectorView<const T\_y> y\_vec(y);

176  VectorView<const T\_loc> mu\_vec(mu);

177  VectorView<const T\_scale> lambda\_vec(lambda);

178  VectorView<const T\_shape> alpha\_vec(alpha);

179  size\_t N = max\_size(y, mu, lambda, alpha);

180

181  agrad::OperandsAndPartials<T\_y, T\_loc, T\_scale, T\_shape>

182  operands\_and\_partials(y, mu, lambda, alpha);

183

184  DoubleVectorView<true, is\_vector<T\_y>::value

185  || is\_vector<T\_loc>::value

186  || is\_vector<T\_scale>::value

187  || is\_vector<T\_shape>::value>

188  p1\_pow\_alpha(N);

189

190  DoubleVectorView<!is\_constant\_struct<T\_y>::value

191  || !is\_constant\_struct<T\_loc>::value

192  || !is\_constant\_struct<T\_scale>::value,

193  is\_vector<T\_y>::value

194  || is\_vector<T\_loc>::value

195  || is\_vector<T\_scale>::value

196  || is\_vector<T\_shape>::value>

197  grad\_1\_2(N);

198

199  DoubleVectorView<!is\_constant\_struct<T\_shape>::value,

200  is\_vector<T\_y>::value

201  || is\_vector<T\_loc>::value

202  || is\_vector<T\_scale>::value

203  || is\_vector<T\_shape>::value>

204  grad\_3(N);

205

206  for (size\_t i = 0; i < N; i++) {

207  const double lambda\_dbl = value\_of(lambda\_vec[i]);

208  const double alpha\_dbl = value\_of(alpha\_vec[i]);

209  const double temp = 1 + (value\_of(y\_vec[i])

210  - value\_of(mu\_vec[i]))

211  / lambda\_dbl;

212  p1\_pow\_alpha[i] = pow(temp, -alpha\_dbl);

213

214  if (!is\_constant\_struct<T\_y>::value

215  || !is\_constant\_struct<T\_loc>::value

216  || !is\_constant\_struct<T\_scale>::value)

217  grad\_1\_2[i] = p1\_pow\_alpha[i] / temp \* alpha\_dbl / lambda\_dbl;

218

219  if (!is\_constant\_struct<T\_shape>::value)

220  grad\_3[i] = log(temp) \* p1\_pow\_alpha[i];

221  }

222

223  // Compute vectorized CDF and its gradients

224

225  for (size\_t n = 0; n < N; n++) {

226

227  // Pull out values

228  const double y\_dbl = value\_of(y\_vec[n]);

229  const double mu\_dbl = value\_of(mu\_vec[n]);

230  const double lambda\_dbl = value\_of(lambda\_vec[n]);

231

232  const double Pn = 1.0 - p1\_pow\_alpha[n];

233

234  // Compute

235  P \*= Pn;

236

237  if (!is\_constant\_struct<T\_y>::value)

238  operands\_and\_partials.d\_x1[n] += grad\_1\_2[n] / Pn;

239  if (!is\_constant\_struct<T\_loc>::value)

240  operands\_and\_partials.d\_x2[n] -= grad\_1\_2[n] / Pn;

241  if (!is\_constant\_struct<T\_scale>::value)

242  operands\_and\_partials.d\_x3[n] += (mu\_dbl - y\_dbl)

243  \* grad\_1\_2[n] / lambda\_dbl / Pn;

244  if (!is\_constant\_struct<T\_shape>::value)

245  operands\_and\_partials.d\_x4[n] += grad\_3[n] / Pn;

246  }

247

248  if (!is\_constant\_struct<T\_y>::value) {

249  for(size\_t n = 0; n < stan::length(y); ++n)

250  operands\_and\_partials.d\_x1[n] \*= P;

251  }

252  if (!is\_constant\_struct<T\_loc>::value) {

253  for(size\_t n = 0; n < stan::length(mu); ++n)

254  operands\_and\_partials.d\_x2[n] \*= P;

255  }

256  if (!is\_constant\_struct<T\_scale>::value) {

257  for(size\_t n = 0; n < stan::length(lambda); ++n)

258  operands\_and\_partials.d\_x3[n] \*= P;

259  }

260  if (!is\_constant\_struct<T\_shape>::value) {

261  for(size\_t n = 0; n < stan::length(alpha); ++n)

262  operands\_and\_partials.d\_x4[n] \*= P;

263  }

264

265  return operands\_and\_partials.to\_var(P);

266  }

267

268  template <typename T\_y, typename T\_loc, typename T\_scale, typename T\_shape>

269  typename return\_type<T\_y, T\_loc, T\_scale, T\_shape>::type

270  pareto\_type\_2\_cdf\_log(const T\_y& y, const T\_loc& mu,

271  const T\_scale& lambda, const T\_shape& alpha) {

272

273  // Check sizes

274  // Size checks

275  if ( !( stan::length(y)

276  && stan::length(mu)

277  && stan::length(lambda)

278  && stan::length(alpha) ) )

279  return 0.0;

280

281  // Check errors

282  static const char\* function = "stan::prob::pareto\_type\_2\_cdf\_log(%1%)";

283

284  using stan::math::check\_greater\_or\_equal;

285  using stan::math::check\_finite;

286  using stan::math::check\_positive\_finite;

287  using stan::math::check\_not\_nan;

288  using stan::math::check\_greater\_or\_equal;

289  using stan::math::check\_consistent\_sizes;

290  using stan::math::check\_nonnegative;

291  using stan::math::value\_of;

292  using stan::math::log1m;

293

294  double P(0.0);

295

296  check\_greater\_or\_equal(function, y, mu, "Random variable", &P);

297  check\_not\_nan(function, y, "Random variable", &P);

298  check\_nonnegative(function, y, "Random variable", &P);

299  check\_positive\_finite(function, lambda, "Scale parameter", &P);

300  check\_positive\_finite(function, alpha, "Shape parameter", &P);

301  check\_consistent\_sizes(function, y, lambda, alpha,

302  "Random variable", "Scale parameter",

303  "Shape parameter", &P);

304

305  // Wrap arguments in vectors

306  VectorView<const T\_y> y\_vec(y);

307  VectorView<const T\_loc> mu\_vec(mu);

308  VectorView<const T\_scale> lambda\_vec(lambda);

309  VectorView<const T\_shape> alpha\_vec(alpha);

310  size\_t N = max\_size(y, mu, lambda, alpha);

311

312  agrad::OperandsAndPartials<T\_y, T\_loc, T\_scale, T\_shape>

313  operands\_and\_partials(y, mu, lambda, alpha);

314

315  DoubleVectorView<true,

316  is\_vector<T\_y>::value

317  || is\_vector<T\_loc>::value

318  || is\_vector<T\_scale>::value

319  || is\_vector<T\_shape>::value>

320  cdf\_log(N);

321

322  DoubleVectorView<true,

323  is\_vector<T\_y>::value

324  || is\_vector<T\_loc>::value

325  || is\_vector<T\_scale>::value

326  || is\_vector<T\_shape>::value>

327  inv\_p1\_pow\_alpha\_minus\_one(N);

328

329  DoubleVectorView<!is\_constant\_struct<T\_shape>::value,

330  is\_vector<T\_y>::value

331  || is\_vector<T\_loc>::value

332  || is\_vector<T\_scale>::value

333  || is\_vector<T\_shape>::value>

334  log\_1p\_y\_over\_lambda(N);

335

336  for (size\_t i = 0; i < N; i++) {

337  const double temp = 1.0 + (value\_of(y\_vec[i])

338  - value\_of(mu\_vec[i]))

339  / value\_of(lambda\_vec[i]);

340  const double p1\_pow\_alpha = pow(temp, value\_of(alpha\_vec[i]));

341  cdf\_log[i] = log1m(1.0 / p1\_pow\_alpha);

342

343  inv\_p1\_pow\_alpha\_minus\_one[i] = 1.0 / (p1\_pow\_alpha - 1.0);

344

345  if (!is\_constant\_struct<T\_shape>::value)

346  log\_1p\_y\_over\_lambda[i] = log(temp);

347  }

348

349  // Compute vectorized CDF and its gradients

350

351  for (size\_t n = 0; n < N; n++) {

352  // Pull out values

353  const double y\_dbl = value\_of(y\_vec[n]);

354  const double mu\_dbl = value\_of(mu\_vec[n]);

355  const double lambda\_dbl = value\_of(lambda\_vec[n]);

356  const double alpha\_dbl = value\_of(alpha\_vec[n]);

357

358  const double grad\_1\_2 = alpha\_dbl

359  \* inv\_p1\_pow\_alpha\_minus\_one[n] / (lambda\_dbl - mu\_dbl + y\_dbl);

360

361  // Compute

362  P += cdf\_log[n];

363

364  if (!is\_constant\_struct<T\_y>::value)

365  operands\_and\_partials.d\_x1[n] += grad\_1\_2;

366  if (!is\_constant\_struct<T\_loc>::value)

367  operands\_and\_partials.d\_x2[n] -= grad\_1\_2;

368  if (!is\_constant\_struct<T\_scale>::value)

369  operands\_and\_partials.d\_x3[n] += (mu\_dbl - y\_dbl) \* grad\_1\_2

370  / lambda\_dbl;

371  if (!is\_constant\_struct<T\_shape>::value)

372  operands\_and\_partials.d\_x4[n] += log\_1p\_y\_over\_lambda[n]

373  \* inv\_p1\_pow\_alpha\_minus\_one[n];

374  }

375

376  return operands\_and\_partials.to\_var(P);

377  }

378

379  template <typename T\_y, typename T\_loc, typename T\_scale, typename T\_shape>

380  typename return\_type<T\_y, T\_loc, T\_scale, T\_shape>::type

381  pareto\_type\_2\_ccdf\_log(const T\_y& y, const T\_loc& mu,

382  const T\_scale& lambda, const T\_shape& alpha) {

383

384  // Check sizes

385  // Size checks

386  if ( !( stan::length(y)

387  && stan::length(mu)

388  && stan::length(lambda)

389  && stan::length(alpha) ) )

390  return 0.0;

391

392  // Check errors

393  static const char\* function = "stan::prob::pareto\_type\_2\_ccdf\_log(%1%)";

394

395  using stan::math::check\_greater\_or\_equal;

396  using stan::math::check\_positive\_finite;

397  using stan::math::check\_not\_nan;

398  using stan::math::check\_greater\_or\_equal;

399  using stan::math::check\_consistent\_sizes;

400  using stan::math::check\_nonnegative;

401  using stan::math::value\_of;

402

403  double P(0.0);

404

405  check\_greater\_or\_equal(function, y, mu, "Random variable", &P);

406  check\_not\_nan(function, y, "Random variable", &P);

407  check\_nonnegative(function, y, "Random variable", &P);

408  check\_positive\_finite(function, lambda, "Scale parameter", &P);

409  check\_positive\_finite(function, alpha, "Shape parameter", &P);

410  check\_consistent\_sizes(function, y, lambda, alpha,

411  "Random variable", "Scale parameter",

412  "Shape parameter", &P);

413

414  // Wrap arguments in vectors

415  VectorView<const T\_y> y\_vec(y);

416  VectorView<const T\_loc> mu\_vec(mu);

417  VectorView<const T\_scale> lambda\_vec(lambda);

418  VectorView<const T\_shape> alpha\_vec(alpha);

419  size\_t N = max\_size(y, mu, lambda, alpha);

420

421  agrad::OperandsAndPartials<T\_y, T\_loc, T\_scale, T\_shape>

422  operands\_and\_partials(y, mu, lambda, alpha);

423

424  DoubleVectorView<true,

425  is\_vector<T\_y>::value

426  || is\_vector<T\_loc>::value

427  || is\_vector<T\_scale>::value

428  || is\_vector<T\_shape>::value>

429  ccdf\_log(N);

430

431  DoubleVectorView<!is\_constant\_struct<T\_y>::value

432  || !is\_constant\_struct<T\_loc>::value

433  || !is\_constant\_struct<T\_scale>::value

434  || !is\_constant\_struct<T\_shape>::value,

435  is\_vector<T\_y>::value

436  || is\_vector<T\_loc>::value

437  || is\_vector<T\_scale>::value

438  || is\_vector<T\_shape>::value>

439  a\_over\_lambda\_plus\_y(N);

440

441  DoubleVectorView<!is\_constant\_struct<T\_shape>::value,

442  is\_vector<T\_y>::value

443  || is\_vector<T\_loc>::value

444  || is\_vector<T\_scale>::value

445  || is\_vector<T\_shape>::value>

446  log\_1p\_y\_over\_lambda(N);

447

448  for (size\_t i = 0; i < N; i++) {

449  const double y\_dbl = value\_of(y\_vec[i]);

450  const double mu\_dbl = value\_of(mu\_vec[i]);

451  const double lambda\_dbl = value\_of(lambda\_vec[i]);

452  const double alpha\_dbl = value\_of(alpha\_vec[i]);

453  const double temp = 1.0 + (y\_dbl - mu\_dbl) / lambda\_dbl;

454  const double log\_temp = log(temp);

455

456  ccdf\_log[i] = -alpha\_dbl \* log\_temp;

457

458  if (!is\_constant\_struct<T\_y>::value

459  || !is\_constant\_struct<T\_loc>::value

460  || !is\_constant\_struct<T\_scale>::value

461  || !is\_constant\_struct<T\_shape>::value)

462  a\_over\_lambda\_plus\_y[i] = alpha\_dbl / (y\_dbl - mu\_dbl + lambda\_dbl);

463

464  if (!is\_constant\_struct<T\_shape>::value)

465  log\_1p\_y\_over\_lambda[i] = log\_temp;

466  }

467

468  // Compute vectorized CDF and its gradients

469

470  for (size\_t n = 0; n < N; n++) {

471  // Pull out values

472  const double y\_dbl = value\_of(y\_vec[n]);

473  const double mu\_dbl = value\_of(mu\_vec[n]);

474  const double lambda\_dbl = value\_of(lambda\_vec[n]);

475

476  // Compute

477  P += ccdf\_log[n];

478

479  if (!is\_constant\_struct<T\_y>::value)

480  operands\_and\_partials.d\_x1[n] -= a\_over\_lambda\_plus\_y[n];

481  if (!is\_constant\_struct<T\_loc>::value)

482  operands\_and\_partials.d\_x2[n] += a\_over\_lambda\_plus\_y[n];

483  if (!is\_constant\_struct<T\_scale>::value)

484  operands\_and\_partials.d\_x3[n] += a\_over\_lambda\_plus\_y[n]

485  \* (y\_dbl - mu\_dbl) / lambda\_dbl;

486  if (!is\_constant\_struct<T\_shape>::value)

487  operands\_and\_partials.d\_x4[n] -= log\_1p\_y\_over\_lambda[n];

488  }

489

490  return operands\_and\_partials.to\_var(P);

491  }

492

493  template <class RNG>

494  inline double

495  pareto\_type\_2\_rng(const double mu,

496  const double lambda,

497  const double alpha,

498  RNG& rng) {

499  static const char\* function = "stan::prob::pareto\_type\_2\_rng(%1%)";

500

501  stan::math::check\_positive(function, lambda, "scale parameter",

502  (double\*)0);

503

504  double uniform\_01 = stan::prob::uniform\_rng(0.0, 1.0, rng);

505

506

507  return (std::pow(1.0 - uniform\_01, -1.0 / alpha) - 1.0) \* lambda + mu;

508  }

509  }

510 }

511 #endif

stan::agrad::log1m

fvar< T > log1m(const fvar< T > &x)

**Definition:** log1m.hpp:16

traits.hpp

stan::prob::pareto\_type\_2\_ccdf\_log

return\_type< T\_y, T\_loc, T\_scale, T\_shape >::type pareto\_type\_2\_ccdf\_log(const T\_y &y, const T\_loc &mu, const T\_scale &lambda, const T\_shape &alpha)

**Definition:** pareto\_type\_2.hpp:381

stan::agrad::OperandsAndPartials::to\_var

T\_return\_type to\_var(double logp)

**Definition:** partials\_vari.hpp:138

stan::math::check\_positive\_finite

bool check\_positive\_finite(const char \*function, const T\_y &y, const char \*name, T\_result \*result)

**Definition:** check\_positive\_finite.hpp:12

uniform.hpp

stan::agrad::pow

fvar< T > pow(const fvar< T > &x1, const fvar< T > &x2)

**Definition:** pow.hpp:17

stan::prob::pareto\_type\_2\_rng

double pareto\_type\_2\_rng(const double mu, const double lambda, const double alpha, RNG &rng)

**Definition:** pareto\_type\_2.hpp:495

error\_handling.hpp

stan::prob::pareto\_type\_2\_cdf\_log

return\_type< T\_y, T\_loc, T\_scale, T\_shape >::type pareto\_type\_2\_cdf\_log(const T\_y &y, const T\_loc &mu, const T\_scale &lambda, const T\_shape &alpha)

**Definition:** pareto\_type\_2.hpp:270

stan::prob::pareto\_type\_2\_cdf

return\_type< T\_y, T\_loc, T\_scale, T\_shape >::type pareto\_type\_2\_cdf(const T\_y &y, const T\_loc &mu, const T\_scale &lambda, const T\_shape &alpha)

**Definition:** pareto\_type\_2.hpp:140

stan::length

size\_t length(const T &)

**Definition:** traits.hpp:159

stan::DoubleVectorView

DoubleVectorView allocates double values to be used as intermediate values.

**Definition:** traits.hpp:358

stan::math::check\_finite

bool check\_finite(const char \*function, const T\_y &y, const char \*name, T\_result \*result)

Checks if the variable y is finite.

**Definition:** check\_finite.hpp:52

partials\_vari.hpp

stan::agrad::value\_of

T value\_of(const fvar< T > &v)

Return the value of the specified variable.

**Definition:** value\_of.hpp:16

constants.hpp

stan::is\_vector

**Definition:** traits.hpp:83

stan::agrad::OperandsAndPartials

A variable implementation that stores operands and derivatives with respect to the variable...

**Definition:** partials\_vari.hpp:76

traits.hpp

stan::return\_type::type

boost::math::tools::promote\_args< typename scalar\_type< T1 >::type, typename scalar\_type< T2 >::type, typename scalar\_type< T3 >::type, typename scalar\_type< T4 >::type, typename scalar\_type< T5 >::type, typename scalar\_type< T6 >::type >::type type

**Definition:** traits.hpp:406

stan::is\_constant\_struct

Metaprogram to determine if a type has a base scalar type that can be assigned to type double...

**Definition:** traits.hpp:57

stan::math::value\_of

double value\_of(const T x)

Return the value of the specified scalar argument converted to a double value.

**Definition:** value\_of.hpp:24

stan::prob::include\_summand

Template metaprogram to calculate whether a summand needs to be included in a proportional (log) prob...

**Definition:** traits.hpp:35

log1m.hpp

stan::agrad::OperandsAndPartials::d\_x2

VectorView< double \*, is\_vector< T2 >::value, is\_constant\_struct< T2 >::value > d\_x2

**Definition:** partials\_vari.hpp:83

stan::prob::uniform\_rng

double uniform\_rng(const double alpha, const double beta, RNG &rng)

**Definition:** uniform.hpp:339

stan::math::check\_nonnegative

bool check\_nonnegative(const char \*function, const T\_y &y, const char \*name, T\_result \*result)

**Definition:** check\_nonnegative.hpp:52

stan::math::check\_consistent\_sizes

bool check\_consistent\_sizes(const char \*function, const T1 &x1, const T2 &x2, const char \*name1, const char \*name2, T\_result \*result)

**Definition:** check\_consistent\_sizes.hpp:13

stan::max\_size

size\_t max\_size(const T1 &x1, const T2 &x2)

**Definition:** traits.hpp:191

stan::math::check\_positive

bool check\_positive(const char \*function, const T\_y &y, const char \*name, T\_result \*result)

**Definition:** check\_positive.hpp:54

stan::math::check\_not\_nan

bool check\_not\_nan(const char \*function, const T\_y &y, const char \*name, T\_result \*result)

Checks if the variable y is nan.

**Definition:** check\_not\_nan.hpp:56

stan::agrad::OperandsAndPartials::d\_x4

VectorView< double \*, is\_vector< T4 >::value, is\_constant\_struct< T4 >::value > d\_x4

**Definition:** partials\_vari.hpp:85

value\_of.hpp

stan::prob::pareto\_type\_2\_log

return\_type< T\_y, T\_loc, T\_scale, T\_shape >::type pareto\_type\_2\_log(const T\_y &y, const T\_loc &mu, const T\_scale &lambda, const T\_shape &alpha)

**Definition:** pareto\_type\_2.hpp:24

stan::agrad::OperandsAndPartials::d\_x1

VectorView< double \*, is\_vector< T1 >::value, is\_constant\_struct< T1 >::value > d\_x1

**Definition:** partials\_vari.hpp:82

stan::agrad::OperandsAndPartials::d\_x3

VectorView< double \*, is\_vector< T3 >::value, is\_constant\_struct< T3 >::value > d\_x3

**Definition:** partials\_vari.hpp:84

constants.hpp

stan::agrad::log

fvar< T > log(const fvar< T > &x)

**Definition:** log.hpp:15

stan::VectorView

VectorView is a template metaprogram that takes its argument and allows it to be used like a vector...

**Definition:** traits.hpp:275

stan::math::check\_greater\_or\_equal

bool check\_greater\_or\_equal(const char \*function, const T\_y &y, const T\_low &low, const char \*name, T\_result \*result)

**Definition:** check\_greater\_or\_equal.hpp:58

stan::agrad::log1p

fvar< T > log1p(const fvar< T > &x)

**Definition:** log1p.hpp:16

stan::math::log1m

boost::math::tools::promote\_args< T >::type log1m(T x)

Return the natural logarithm of one minus the specified value.

**Definition:** log1m.hpp:40


---

[ Stan Home Page ]

*© 2011–2014,
Stan Development Team.*
